# Supplementary material for: Development of a Tool to Assess the Implementation and Perception of the Value-Based Healthcare Model: Our Results
Source: Healthcare (Basel). 2025 Nov 10;13(22):2849. doi: 10.3390/healthcare13222849 (PMC12652851; doi:10.3390/healthcare13222849)
Supplement: Supplementary file 1 [file healthcare-13-02849-s001.zip › healthcare-3931176-supplementary.pdf]

	Mean	SD	CV	Median	IQR	RIR	Kendall's W	Mean	SD	CV	Median	IQR	RIR	Kendall's W	VRIR	W_statistic	p_value	Consensus
**VALUE AGENDA SECTION**																		
N°1	4.56	0.821	0.18	5	1.00	0.20	W = 0.196 ***	4.35	0.775	0.18	5	1.00	0.20	W = 0.222***	0.00	340	0.213	A
N°2	4.40	0.913	0.21	5	1.00	0.20		4.57	0.788	0.17	5	0.50	0.10		0.10	254.5	0.421	A
N°3	4.56	0.712	0.16	5	1.00	0.20		4.74	0.619	0.13	5	0.00	0.00		0.20	247.5	0.282	A
N°4	4.52	0.872	0.19	5	1.00	0.20		4.91	0.288	0.06	5	0.00	0.00		0.20	228	0.073	A
N°5	4.44	0.768	0.17	5	1.00	0.20		4.65	0.714	0.15	5	0.50	0.10		0.10	236	0.210	A
N°6	4.84	0.374	0.08	5	0.00	0.00		4.78	0.518	0.11	5	0.00	0.00		0.00	293.5	0.860	A
N°7	4.72	0.542	0.11	5	0.00	0.00		4.61	0.583	0.13	5	1.00	0.20		-0.20	317.5	0.442	A
N°8	4.60	0.500	0.11	5	1.00	0.20		4.48	1.082	0.24	5	0.50	0.10		0.10	262.5	0.541	A
N°9	4.68	0.690	0.15	5	0.00	0.00		4.74	0.541	0.11	5	0.00	0.00		0.00	287.5	1	A
N°10	4.32	0.900	0.21	5	1.00	0.20		4.52	0.665	0.15	5	1.00	0.20		0.00	261	0.543	A
N°11	4.76	0.523	0.11	5	0.00	0.00		4.78	0.518	0.11	5	0.00	0.00		0.00	280.5	0.843	A
N°12	4.48	0.770	0.17	5	1.00	0.20		4.70	0.559	0.12	5	0.50	0.10		0.10	251.5	0.369	A
N°13	4.48	0.823	0.18	5	1.00	0.20		4.61	0.583	0.13	5	1.00	0.20		0.00	276	0.787	A
	**Mean**	**SD**	**CV**	**Median**	**IQR**	**RIR**		**Mean**	**SD**	**CV**	**Median**	**IQR**	**RIR**		**VRIR**	**Mann-Whitney U (W_statistic)**	**Mann-Whitney U (p_value)**	**Consensus**
**CHANGE MANAGEMENT SECTION**																		
N°14	4.40	0.957	0.22	5	1.00	0.20	W = 0.208 ***	4.70	0.559	0.12	5	0.50	0.1	W = 0.28***	0.10	250.5	0.356	A
N°15	4.48	0.918	0.20	5	1.00	0.20		4.65	0.487	0.10	5	1.00	0.2		0.00	283.5	0.930	A
N°16	4.60	0.500	0.11	5	1.00	0.20		4.57	0.590	0.13	5	1.00	0.2		0.00	290	0.961	A
N°17	4.60	0.577	0.13	5	1.00	0.20		4.43	0.945	0.21	5	1.00	0.2		0.00	301.5	0.743	A
N°18	4.40	0.816	0.19	5	1.00	0.20		4.43	0.662	0.15	5	1.00	0.2		0.00	292	0.926	A
N°19	4.56	0.768	0.17	5	1.00	0.20		4.57	0.507	0.11	5	1.00	0.2		0.00	310.5	0.584	A
N°20	4.60	0.764	0.17	5	1.00	0.20		4.52	0.665	0.15	5	1.00	0.2		0.00	316.5	0.478	A
N°21	4.76	0.663	0.14	5	0.00	0.00		4.48	0.898	0.20	5	1.00	0.2		-0.20	352	0.088	A
N°22	4.60	0.764	0.17	5	1.00	0.20		4.65	0.885	0.19	5	0.00	0.00		0.20	269	0.623	A
N°23	4.36	0.860	0.20	5	1.00	0.20		4.43	0.590	0.13	4	1.00	0.25		-0.05	292	0.926	A
N°24	4.60	0.645	0.14	5	1.00	0.20		4.57	0.728	0.16	5	1.00	0.2		0.00	288	1	A
N°25	4.76	0.436	0.09	5	0.00	0.00		4.78	0.422	0.09	5	0.00	0.000		0.00	281	0.865	A
N°26	4.56	0.651	0.14	5	1.00	0.20		4.65	0.573	0.12	5	1.00	0.2		0.00	269	0.653	A
N°27	4.84	0.374	0.08	5	0.00	0.00		4.74	0.541	0.11	5	0.00	0.00		0.00	306	0.583	A

N°28	4.64	0.569	0.12	5	1.00	0.20	4.70	0.635	0.14	5	0.00	0.00	0.20	263.5	0.532	A
N°29	4.44	0.821	0.18	5	1.00	0.20	4.70	0.703	0.15	5	0.00	0.00	0.20	230	0.141	A
N°30	4.84	0.374	0.08	5	0.00	0.00	4.70	0.876	0.19	5	0.00	0.00	0.00	293.5	0.861	A
